# Complete Genome Sequence of *Rice hoja blanca tenuivirus* Isolated from a Susceptible Rice Cultivar in Colombia

**DOI:** 10.1128/genomeA.01490-17

**Published:** 2018-02-15

**Authors:** Jenyfer Jimenez, Monica Carvajal-Yepes, Ana Maria Leiva, Maribel Cruz, Luz Elena Romero, Carmen A. Bolaños, Ivan Lozano, Wilmer J. Cuellar

**Affiliations:** a Virology Laboratory, Agrobiodiversity Research Area (AgBio), International Center for Tropical Agriculture (CIAT), Cali, Colombia; b Facultad de Ciencias Agropecuarias, Universidad Nacional de Colombia (UNAL), Palmira, Colombia; c Latin American Fund for Irrigated Rice (FLAR), Cali, Colombia

## Abstract

We describe here the complete genome of *Rice hoja blanca tenuivirus*. The sequenced isolate was obtained by insect vector transmission from a symptomatic rice sample grown in Colombia. Sequence data from the four RNA components were obtained by deep sequencing (Illumina), and infections were confirmed by enzyme-linked immunosorbent assay and Sanger sequencing.

## GENOME ANNOUNCEMENT

*Rice hoja blanca tenuivirus* (RHBV) is a multipartite single-stranded, negative-sense RNA virus belonging to the genus *Tenuivirus*. It causes hoja blanca disease (HBD) in rice (*Oryza sativa* L.), which has been reported as affecting rice yield in Colombia since 1935. The virus was first described in 1983 (1) and partially characterized between 1992 and 1993 (2, 3). Since then, HBD has been reported in countries of major rice production in Latin America, the Caribbean, and the southern United States. The vector of RHBV is *Tagosodes orizicolus* Müir (Hemiptera: Delphacidae), which can also be infected by the virus (4). Infected rice plants show symptoms that include chlorotic striping and mottling on the leaves, plant dwarfing, panicle sterility, premature wilting, and necrosis, causing serious yield losses (25 to 75%; [4]). Until now, a complete genome sequence of RHBV has not been available.

To obtain the complete genome of RHBV, the rice cultivar Bluebonnet 50 was infected with an isolate from Tolima (central Colombia) using *T. orizicolus* as a vector. Total RNA extraction was carried out, using TRIzol (5), from 50 mg of dried leaf tissue. cDNA libraries were prepared from small interfering RNAs (siRNAs) as previously described (6), and deep sequencing was performed using the Illumina (San Diego, CA, USA) platform, according to the manufacturer’s instructions. Removal of the 3′ end adapters resulted in 1,676,078 reads between 21 and 24 nucleotides (nt) in length that generated 162 contigs; the conditions for contig assembly were a minimal overlapping length *k*-mer of 15 and minimum contig length of 50 nt. Genome assembly was achieved using Velvet version 1.2.03 for *de novo* sequence assembly (7) and MAQ version 0.7.1 software (http://maq.sourceforge.net). Compared to the GenBank nonredundant database using BLASTx and BLASTn searches, 33.33% of the total number of contigs showed significant similarity with different viral sequences reported for RHBV. RHBV encodes a typical tenuivirus genome arrangement compared to *Rice stripe tenuivirus* (RSV), the type species of the group (8). It is composed of four RNA components, where RNA2 to RNA4 encode two open reading frames (ORFs), the second of which is encoded by the complementary strand. All RNA components present a conserved complementary 5′ and 3′ terminal nucleotide sequence (5′ ACACAAAGTC 3′). RHBV RNA1 (8,999 bp) encodes the RNA-dependent RNA polymerase (RdRp; 73%). RNA2 (3,620 bp) has an ambisense tanslation strategy encoding a nonstructural (NS2; 60%) protein in the 5′ proximal end and the membrane glycoprotein (68%) at the 3′ proximal ORF. RNA3 (2,299 bp) also encodes two ORFs, the NS3 protein (67%) in the 5′ proximal ORF and the nucleocapsid protein (65%) in the 3′ proximal ORF. RNA4 (1,998 bp) encodes the major nonstructural protein (NCP; 74%) at the 5′ proximal end and the NS4 protein (75%) at the 3′ proximal end. The percentage values indicate the amino acid sequence similarity to RSV for each RHBV protein. Specific primers and Sanger sequencing (Macrogen, South Korea) were used to confirm the assembled sequences and filling in of gapped regions.

### Accession number(s).

The complete genome sequences reported here have been deposited at GenBank under the accession no. MG566074 (RNA1), MG566075 (RNA2), MG566076 (RNA3), and MG566077 (RNA4).
